# Autonomic Nervous Dysfunction and Ultra-Short-Term Heart Rate Variability in Atrial Fibrillation: Recent Advances in Early Detection

**DOI:** 10.3390/jcdd13060286

**Published:** 2026-06-22

**Authors:** Shanquan Gao, Xiaodi Tang

**Affiliations:** Beijing Tsinghua Changgung Hospital, School of Clinical Medicine, Tsinghua Medicine, Tsinghua University, No. 168 Litang Road, Changping District, Beijing 102218, China; gsqa03621@btch.edu.cn

**Keywords:** atrial fibrillation, autonomic nervous function, heart rate variability, ultra-short-term heart rate variability

## Abstract

The development of atrial fibrillation involves the synergistic effects of electrical remodeling, structural remodeling and neural remodeling, among which remodeling of the autonomic nervous system (ANS) plays a pivotal role in disease initiation and progression. Heart rate variability (HRV), as an important tool for assessing autonomic function, has been widely applied in cardiovascular research. In particular, ultra-short-term heart rate variability (usHRV) analysis has demonstrated significant value in the early prediction of atrial fibrillation.

## 1. Autonomic Nervous System Function and Neural Remodelling

The initiation and maintenance of atrial fibrillation (AF) involve a complex interplay of electrical, structural, and neural remodelling—collectively the “triple-remodelling theory”. These processes interact and reinforce one another, forming the pathophysiological substrate for AF [1]. Within this framework, the autonomic nervous system (ANS) plays a pivotal role. Neural remodelling directly influences atrial electrophysiological properties and interacts closely with electrical and structural remodelling, thereby affecting AF initiation, maintenance, and clinical outcomes [2,3].

### 1.1. Distribution of the Cardiac Autonomic Nervous System

The cardiac ANS comprises extrinsic and intrinsic components that maintain cardiac rhythm through sympathovagal balance. The extrinsic ANS includes sympathetic fibers from paravertebral ganglia (notably the stellate ganglion) and parasympathetic fibers carried mainly by the vagus nerve. The stellate ganglion is a dominant source of cardiac sympathetic innervation. Most of its neurons are sympathetic, but a small subset (~5%) exhibit a cholinergic phenotype. Co-expression of both phenotypes has been observed, and such phenotypic switching may occur under disease conditions or after neuromodulation [4].

Extrinsic parasympathetic fibers travel via the vagus nerve, converging at a fat pad between the superior vena cava and aorta before reaching the sinus and AV nodes. Of note, the vagus nerve is not purely parasympathetic; sympathetic fibers comprise approximately 1–5% of its cross-sectional area. The intrinsic cardiac ANS consists of ganglionated plexi (GP) embedded in epicardial fat pads and myocardium. These GP contain heterogeneous neuronal populations—predominantly local circuit neurons—along with sympathetic, parasympathetic, and afferent neurons. Serving as integrative centers, GP modulate interactions between extrinsic and intrinsic ANS through cardio-cardiac reflexes [5].

Regional heterogeneity is a hallmark of cardiac ANS. The sinus node and AV node receive the densest innervation: the sinus node is predominantly innervated by right-sided fibers, whereas the AV node is governed by left-sided fibers. Atrial myocardium is predominantly under vagal control, with higher vagal density in the atrial appendages. In the ventricles, sympathetic fibers are largely confined to the epicardial surface, while vagal fibers are sparse [6]. Within the atria, GP are concentrated at distinct sites, particularly the pulmonary vein–left atrial junction. Five major GP have been consistently described: right atrial GP, inferior vena cava–inferior atrial GP, left inferior GP, left superior GP, and the ligament of Marshall tract. This anatomic heterogeneity provides the substrate for region-specific autonomic modulation and has implications for arrhythmogenesis and therapy [7].

### 1.2. Electrophysiological Mechanisms of Sympathetic Activation in Atrial Fibrillation

Sympathetic activation promotes AF through multiple pathways. Norepinephrine binds to myocardial β-adrenergic receptors, triggering the Gs-cAMP-PKA cascade. PKA-dependent phosphorylation enhances L-type calcium current (ICaL) and increases sarcoplasmic reticulum calcium release via RyR2 phosphorylation. The resulting calcium overload facilitates delayed afterdepolarizations (DADs) via NCX-mediated transient inward currents, increasing triggered activity and focal ectopic firing [8].

Sympathetic excitation also modulates potassium channels involved in repolarization, enhancing IKur and Ito, which accelerate early- and mid-phase repolarization. These alterations increase spatial heterogeneity of repolarization. Under conditions of prolonged action potential duration (APD)—e.g., long QT syndrome, bradycardia, hypokalemia, or class III antiarrhythmic drugs—sympathetic activation can promote early afterdepolarizations (EADs). Mechanistically, enhanced ICaL during the plateau phase reactivates L-type channels, generating a depolarizing current that interrupts repolarization. This EAD-mediated triggered activity is particularly relevant to AF in patients with long QT syndrome, in whom sympathetic surges precipitate torsade de pointes and AF [9].

Sympathetic nerve fibers are heterogeneously distributed across the atria, with higher densities in the posterior left atrium and pulmonary vein region. This leads to regionally unequal shortening of refractoriness following sympathetic activation, increasing spatial dispersion of effective refractory periods and creating a substrate for unidirectional block and re-entry. Collectively, these sympathetic mechanisms—enhanced triggered activity (via DADs/EADs), increased repolarization heterogeneity, and re-entry substrate formation—synergistically promote AF susceptibility and persistence [10].

### 1.3. Electrophysiological Mechanisms of Vagal Activation in AF

Vagal nerve fibers are heterogeneously distributed across the atria, leading to regionally divergent responses to vagal stimulation. This increases spatial dispersion of refractoriness and promotes functional re-entry. At the molecular level, acetylcholine activates M2 receptors, opening IKACh channels, which shortens atrial APD and ERP. This abbreviation increases local electrophysiological heterogeneity, promotes micro-re-entry, and reduces re-entrant wavelength, thereby facilitating AF maintenance [11].

Enhanced vagal activity also increases atrial electrophysiological heterogeneity, promoting focal ectopic drivers via enhanced automaticity, EADs, and DADs. Scherlag and colleagues demonstrated that acetylcholine from cardiac GP preferentially shortens ERP at the atrial myocardium and pulmonary vein sleeves. Concurrent sympathetic co-activation amplifies the proarrhythmic milieu: adrenergic neurotransmitters elevate intracellular calcium, and a prolonged early repolarization state with extended calcium release leads to cytosolic calcium accumulation. This generates a net inward current (via ~3:1 sodium influx/calcium efflux ratio) that triggers early repolarization and triggered activity, culminating in AF initiation. Clinical studies confirm that thoracoscopic epicardial ablation of left atrial GP is effective in AF treatment [12].

Enhanced vagal tone exerts a negative chronotropic effect on the sinus node via M2 receptor activation and IKACh, hyperpolarizing pacemaker cells and suppressing diastolic depolarization. Physiological slowing is reversible, but excessive vagal activity can cause sinus bradycardia, block, or arrest. This suppression creates a substrate for ectopic foci via two mechanisms. First, prolonged diastolic interval allows latent pacemakers (atria, pulmonary veins, AV junction) to reach threshold spontaneously (“overdrive suppression release”). Second, vagally mediated heterogeneity in atrial repolarization increases dispersion of refractoriness, favoring unidirectional block and re-entry [9]. These changes transform the quiescent atrium into a tissue capable of generating premature complexes and initiating paroxysmal AF, with important clinical implications for vagally mediated AF and nocturnal bradycardia-dependent arrhythmias [13].

### 1.4. Sympathetic–Vagal Interaction

Autonomic regulation of the atria involves complex sympathetic–parasympathetic interactions. Vagal innervation exhibits spatial heterogeneity, leading to regional differences in neural responses. During AF initiation, this balance is disrupted, with concomitant activation of both branches and diminished inhibitory interaction. Co-activation shortens atrial APD while prolonging Ca^2+^ transients, creating a substrate for phase 3 early afterdepolarizations in intermediate/late AF stages. Shortened APD also promotes re-entry, particularly in the pulmonary vein region, an area prone to short APD and Ca^2+^-induced arrhythmogenesis [12]. Medical interventions such as radiofrequency ablation and renal sympathetic denervation attenuate atrial autonomic innervation and reduce arrhythmia risk [14]. This mechanistic link supports the conclusions of the present study.

Simultaneous sympathetic and vagal activation at AF onset is accompanied by attenuation of their normal inhibition. Combined stimulation synergistically induces atrial tachyarrhythmias [15]. Parasympathetic activation increases spatial heterogeneity and dispersion, promoting micro-re-entry, while sympathetic activation induces triggered activity via elevated intracellular Ca^2+^. Thus, concurrent autonomic activation promotes AF through enhanced triggered activity and re-entry [5,16].

Neural remodelling patterns vary by AF subtype and duration. In paroxysmal AF, remodelling is mild, with modest nerve density increases mainly around the pulmonary veins. Autonomic dysfunction is intermittent, most evident before and during AF episodes. Most patients exhibit increased vagal tone, with AF episodes often occurring at rest or night. In contrast, persistent AF involves more extensive remodelling across larger atrial areas, often with a sympathetic-dominant pattern that may become irreversible. Thus, pathological autonomic alterations may persist even after sinus rhythm restoration [12,17].

## 2. Heart Rate Variability and Autonomic Regulation

Heart rate variability (HRV) is a widely used non-invasive method for assessing cardiac autonomic function. Its importance in AF research has increased substantially in recent years. HRV provides quantitative information regarding the relative activity of sympathetic and parasympathetic systems and reflects the overall balance of autonomic regulation. As such, HRV analysis has become an essential tool for studying cardiovascular autonomic function [18].

HRV refers to the small fluctuations in time intervals between consecutive heartbeats. Physiologically, these variations reflect dynamic modulation of the sinoatrial node by the autonomic nervous system. As a result, adjacent RR intervals during normal sinus rhythm typically differ by several to several tens of milliseconds. By analysing patterns of RR interval variation, HRV can be used to evaluate autonomic control of cardiac rhythm and provide insights into autonomic tone and physiological adaptability. HRV indices are generally derived from RR interval sequences recorded on electrocardiography [19].

### 2.1. Overview of Heart Rate Variability Indices

According to the recommendations of the Task Force of the European Society of Cardiology and the North American Society of Pacing and Electrophysiology, HRV analysis can be broadly categorized into time-domain, frequency-domain, and nonlinear analyses, all derived from normal-to-normal (NN) interval series [20].

Time-domain indices mainly include the standard deviation of NN intervals (SDNN), standard deviation of successive differences (SDSD), root mean square of successive differences (RMSSD), percentage of successive NN interval differences greater than 50 ms [pNN50 (%)], and standard deviation of the averages of NN intervals (SDANN).

Frequency-domain measures quantify spectral power within predefined frequency bands, including very low frequency power (VLF, 0.003–0.04 Hz), low frequency power (LF, 0.04–0.15 Hz), and high frequency power (HF, 0.15–0.40 Hz). Normalized indices—LF in normalized units [LF(nu)] and HF in normalized units [HF(nu)]—as well as the LF/HF ratio are commonly used to assess the relative balance between sympathetic and parasympathetic modulation [21].

In addition, nonlinear HRV analysis evaluates the complex and dynamic properties of heart rate regulation that cannot be fully captured by linear methods. Common nonlinear approaches include Poincaré plot analysis, approximate entropy, sample entropy, and detrended fluctuation analysis, which provide complementary information regarding autonomic regulation and cardiovascular dynamics [22].

### 2.2. Time-Domain Measures

Time-domain analysis represents the most straightforward and intuitive approach for evaluating HRV. The principal statistical indices include SDNN, which reflects overall variability in heart rate and represents the combined influence of both slow regulatory processes—such as sympathetic activity, circadian rhythms, and thermoregulation—and rapid fluctuations associated with respiration. SDSD and RMSSD, which are calculated from differences between adjacent NN intervals, primarily capture short-term, high-frequency fluctuations in heart rate. Because these indices are derived from successive interval differences, they effectively filter out slower trends and are therefore considered sensitive markers of parasympathetic (vagal) modulation of cardiac activity. pNN50 (%) represents the proportion of successive NN interval pairs that differ by more than 50 ms and is commonly used as an indicator of vagal activity. SDANN, calculated as the standard deviation of the average NN intervals across 5-min segments, reflects long-term components of HRV and characterizes overall variability over extended time scales [20].

### 2.3. Frequency-Domain Measures

Frequency-domain analysis transforms time-domain NN interval series into the frequency domain using fast Fourier transformation (FFT). This approach focuses on spectral power within several characteristic frequency bands. VLF (0.003–0.04 Hz) primarily reflects long-term regulatory mechanisms and nonlinear characteristics of heart rate dynamics and has been associated with physiological processes such as thermoregulation, activity of the renin–angiotensin–aldosterone system, and vasomotor function. LF (0.04–0.15 Hz) reflects the combined influence of sympathetic and parasympathetic modulation but is generally considered to be more strongly influenced by sympathetic activity, particularly during conditions such as standing, physical exertion, or psychological stress. LF also reflects oscillations related to baroreflex regulation. LF (nu) represents the normalized value of LF after removal of the influence of total power and the vLF component. This index reflects the relative contribution of sympathetic modulation to overall autonomic balance. HF (0.15–0.40 Hz) primarily reflects vagal activity and is closely associated with respiratory rhythms; therefore, this frequency band is often referred to as the respiratory band. During inspiration, heart rate typically increases (resulting in a decrease in HF power), whereas during expiration heart rate decreases (resulting in an increase in HF power). HF (nu) represents the normalized value of HF after removal of the influence of total power and VLF, thereby providing a more accurate estimate of the relative contribution of vagal modulation to autonomic balance. The LF/HF ratio is widely used to assess the relative balance between sympathetic and parasympathetic activity [23].

### 2.4. Nonlinear Analysis

Nonlinear analysis represents a rapidly developing area in HRV research and includes several important analytical approaches. Poincaré plot analysis characterizes the dynamic fluctuations of NN intervals based on scatter plot patterns. Fractal dimension analysis is used to quantify the complexity of HRV signals. Approximate entropy measures the irregularity and unpredictability of HRV time series. In addition, detrended fluctuation analysis (DFA) is commonly applied to determine whether long-range correlations exist within heart rate variability signals [22].

### 2.5. Role of Heart Rate Variability in Atrial Fibrillation Prediction

HRV is an important indicator of autonomic nervous system activity. The interplay between sympathetic and parasympathetic regulation determines the pattern of HRV fluctuations. Abnormal HRV patterns are closely associated with the pathophysiological processes of various cardiovascular diseases. For example, reduced HRV in patients with coronary artery disease correlates with both the extent and severity of atherosclerotic lesions and may serve as an independent predictor of disease progression. Alterations in HRV are also associated with hypertension, heart failure, diabetes, and other cardiovascular disorders [24].

The ANS plays a critical role in the initiation and recurrence of AF. As a marker of cardiac autonomic activity, HRV offers a non-invasive and sensitive method for monitoring patients with AF. Reduced HRV may indicate an increased risk of AF onset or recurrence [25].

Recent studies have further demonstrated the predictive value of HRV. Among patients undergoing cardiac surgery, lower preoperative HRV levels are associated with a significantly increased risk of postoperative AF [26]. In particular, SDNN values below 50 ms have been identified as an independent predictor of postoperative AF. Similarly, in patients undergoing catheter ablation for AF, abnormal preoperative HRV has been linked to a higher risk of AF recurrence after the procedure. HRV analysis may therefore assist in identifying high-risk individuals and guiding preventive and therapeutic strategies [27]. In high-risk populations, such as individuals with hypertension, abnormal HRV is also associated with an increased risk of incident AF.

Although the development of AF involves multiple factors—including male sex, obesity, obstructive sleep apnea, alcohol consumption, endurance exercise, and family history—clinical studies indicate that most cases of isolated paroxysmal AF display a clear vagal-dependent pattern [28]. In a study by Lo et al. involving 30 patients with paroxysmal AF, vagal-dependent AF was characterized by increased left atrial myocardial voltage, shortened excitation duration, and reduced atrial volume, whereas sympathetic-dependent AF exhibited enlarged left atrial volume, a greater number of extra-pulmonary vein triggers, and a significantly higher post-ablation recurrence rate [29].

Although PET/CT has been widely used, techniques for directly assessing ANS activity remain limited and require further empirical and technical support [30]. Clinically, the classification of sympathetic- versus vagal-dependent AF primarily relies on 24-h ambulatory ECG HRV frequency-domain analysis: an increase in HF accompanied by a reduction in the LF/HF ratio indicates vagal-dependent AF, whereas an elevated LF/HF ratio suggests sympathetic-dependent AF. It should be noted that the activities of both autonomic branches are often simultaneously activated in many circumstances. Current measurement techniques are insufficient to quantitatively assess the intensity and interaction of sympathetic and parasympathetic activation in the heart. Consequently, there remains a lack of robust experimental methods to elucidate the specific roles of sympathetic and vagal components of the ANS in AF pathogenesis [3].

Evidence from prior studies suggests that sympathetic and parasympathetic activity exert distinct influences on atrial electrophysiology. These two branches can independently trigger AF, but may also act synergistically; in either scenario, the pre-existing sympathetic–parasympathetic balance is disrupted [31]. Recently, the concept of “autonomic imbalance” has been proposed to explain atrial electrical instability and AF onset resulting from stress on different autonomic components. This theory posits that activation, or preferential activation, of any component of the ANS can induce a sympathetic–parasympathetic imbalance, and that achieving “rebalancing” requires quantitative evaluation of both the intensity and timing of activation across different ANS components [3]. However, research on this topic remains limited, and elucidating these mechanisms is expected to become a key focus in the development of future AF prevention and management strategies [32].

### 2.6. Ultra-Short Heart Rate Variability as an Emerging Predictive Marker

Traditional HRV analysis generally requires relatively long ECG recordings, such as 5-min or 24-h monitoring, and relies on stable signal quality. These requirements limit its applicability in real-time clinical monitoring settings [32]. Ultra-short heart rate variability (usHRV) analysis enables extraction of HRV information from short ECG recordings ranging from 10 s to 2 min, offering substantial practical advantages in clinical settings [33,34]. UsHRV provides rapid assessment, requires minimal recording time, and allows repeated measurements, making it well suited for real-time monitoring and diverse clinical environments [35,36]. The prognostic value of reduced HRV derived from longer recordings has been validated in several population-based cohorts, including ARIC [37], Framingham [38] and MESA [39], and further supported by a meta-analysis of eight studies [40].

Furthermore, several studies have demonstrated strong agreement between usHRV and conventional HRV metrics. For example, Professor Michele Orini’s team examined 1337 participants in the UK Biobank and confirmed a high degree of agreement between usHRV and conventional HRV metrics [33]. Research has demonstrated excellent agreement between RMSSD and SDSD derived from ultra-short-term (15 s) and short-term (6 min) heart rate variability recordings. HRV measured from recordings shorter than 15 s was associated with increased long-term risks of atrial fibrillation, major adverse cardiovascular events (MACE), stroke and mortality, independent of resting heart rate and conventional cardiovascular risk factors. In adjusted models, reductions in usHRV were associated with long-term risks of atrial fibrillation, MACE and mortality, with effect sizes comparable to established risk factors such as diabetes, hypertension and smoking. RMSSD and SDSD derived from 10-s or 5-s electrocardiograms showed similar prognostic associations, supporting the potential of usHRV as a preclinical biomarker for multiple clinical outcomes.

Another study by Professor L. Pecchia involved collecting ECG data from 42 subjects under both stress and resting conditions. The study extracted five HRV features with varying durations and utilized non-parametric tests, correlation analysis, and machine learning algorithms such as IBK to compare HRV feature differences between stress and resting states. The correlation between usHRV and HRV was assessed, and models were trained and tested for classification performance. The results indicated that of the 23 HRV features, six (MeanNN, StdNN, MeanHR, StdHR, HF, SD2) demonstrated consistency within the 1–5 min duration range, exhibiting significantly aligned trends and high correlation with short-term features. Three features (MeanNN, StdHR, HF) showed stress detection accuracy exceeding 88% when incorporated into machine learning models. The IBK-based model achieved optimal performance using 3-min HRV data (AUC 97%, accuracy 94%), while still maintaining strong performance with 1-min data (accuracy 88%) [35].

Professor Harold Snieder quantified SDNN and RMSSD from ultra-short and short ECG recordings of 10 s, 30 s, and 120 s in 3387 adults, and compared these measurements with those derived from 240–300 s recordings (gold standard). Consistency was evaluated using the Pearson correlation coefficient (r), Bland–Altman 95% limits of agreement, and Cohen’s d statistics. The findings demonstrated high agreement between single 10-s recordings and 240–300 s recordings (SDNN: r = 0.758–0.764; RMSSD: r = 0.853–0.862). Averaging SDNN and RMSSD across three 10-s segments further improved agreement with the gold standard (SDNN: r = 0.863; RMSSD: r = 0.941). Consistency increased with recording duration, approaching near-perfect agreement for 120-s recordings (SDNN: r = 0.956; RMSSD: r = 0.986). Across all recording durations, RMSSD consistently demonstrated stronger agreement than SDNN [41].

However, methodological considerations remain important for optimizing usHRV implementation. A detailed evaluation by Burma and colleagues confirmed that while ultra-short recordings inherently involve some trade-offs, several usHRV metrics still demonstrate acceptable validity and reliability under standardized conditions. Specifically, heart rate and key time-domain parameters (e.g., RMSSD) can be reliably derived from recordings as short as 60–240 s, with progressively better performance at longer durations. Importantly, the observed variability in usHRV was largely attributable to measurable physiological confounders (e.g., respiratory rate, blood pressure), which can be controlled in clinical practice [34]. These findings do not undermine the utility of usHRV but rather guide its appropriate application: for rapid screening or repeated measurements in settings such as atrial fibrillation risk assessment, well-selected usHRV parameters from 1–2 min recordings offer a practical and sufficiently reliable alternative to conventional HRV. To provide a more comprehensive understanding of the relationship between usHRV, AF, and HRV, we systematically searched the literature and now present a summary of the relevant studies in Supplementary Table S1.

In the context of AF early warning, usHRV analysis demonstrates several notable advantages. Its real-time monitoring capability allows continuous tracking and timely detection of abnormal fluctuations in ANS activity. Its dynamic assessment function enables uninterrupted monitoring to capture temporal trajectories of autonomic regulation [42]. Furthermore, individualized prediction can be enhanced by constructing customized models, thereby significantly improving predictive accuracy [43]. Artificial intelligence offers substantial potential in HRV analysis: machine learning algorithms can efficiently identify complex patterns within HRV data, enhancing prediction reliability, while deep learning models are capable of processing large-scale HRV datasets to uncover subtle, higher-order patterns that are difficult to detect manually [44].

### 2.7. Future Research Directions for Ultra-Short-Term Heart Rate Variability

Compared with conventional HRV, usHRV offers advantages of convenient acquisition, enhanced interference resistance, and broader clinical applicability. However, research utilizing usHRV for AF risk assessment across diverse populations remains limited globally. UsHRV analysis demonstrates significant strengths in AF early warning: its real-time monitoring capability enables continuous tracking and prompt detection of abnormal autonomic nervous fluctuations; its dynamic assessment capacity facilitates real-time analysis of autonomic function trajectories through uninterrupted monitoring; and its personalized alert mechanism substantially improves predictive accuracy via customized modeling [45]. Artificial intelligence holds considerable promise in HRV analysis: machine learning algorithms can efficiently identify complex HRV patterns to enhance prediction reliability, while deep learning models possess the capability to process large-scale usHRV data, uncovering subtle patterns and regularities imperceptible to human vision.

#### 2.7.1. Deepening the Clinical Application of Ultra-Short-Term Heart Rate Variability: From Static Snapshot to Dynamic Trajectories

Current studies predominantly rely on single or intermittent usHRV measurements, which may overlook the critical information embedded in the temporal evolution of autonomic tone. Future research should prioritize investigating the dynamic trends of usHRV over hours to days preceding AF onset. Specifically, we hypothesize that a progressive decline in parasympathetic indices (e.g., RMSSD) combined with a fluctuating sympathovagal balance may constitute a “proarrhythmic trajectory” detectable 6–24 h before AF initiation. Rather than isolated thresholds, the rate, magnitude, and temporal pattern of autonomic changes—captured via continuous monitoring—are likely to yield superior predictive value [46].

Integrating usHRV into consumer-grade wearables (e.g., smartwatches, chest patches) and smartphone-enabled single-lead electrocardiograms represents a logical and urgently needed next step. Key research questions include: What is the minimal acceptable recording duration and quality required for reliable usHRV trend analysis under free-living conditions? How should physical activity, sleep stages, and emotional states be modeled as confounders? Validation of real-world risk models using long-term, home-based usHRV data—ideally through prospective multicenter trials—will be essential before clinical adoption [47].

#### 2.7.2. Artificial Intelligence and Multi-Omics Integration

The subtle, nonlinear, and high-dimensional patterns embedded in long-sequence usHRV data are largely beyond human visual recognition or traditional linear statistics. We advocate for the application of advanced machine learning (e.g., gradient boosting, random forests) and deep learning architectures (e.g., transformers, convolutional long short-term memory networks, graph neural networks) to decode these complex autonomic fingerprints for individualized AF prediction. Unlike conventional risk scores, these models can capture time-dependent interactions among multiple HRV metrics without prespecified features. Future work should also address model explainability. For instance, using Shapley additive explanations or attention mechanisms to identify which usHRV patterns drive predictions, thereby enhancing clinical trust [48].

Furthermore, a paradigm shift toward multi-omics integration is essential. Combining usHRV-derived autonomic indices with genomics (e.g., single-nucleotide polymorphisms in cholinergic or adrenergic pathway genes, such as *CHRM2* or *ADRB1*), proteomics (e.g., circulating neuropeptide Y, galanin, or inflammatory cytokines), and metabolomics (e.g., short-chain fatty acids modulating autonomic outflow), could unmask novel molecular signatures of autonomic dysfunction. Future studies should adopt a discovery-validation design: first, unsupervised clustering of long-term usHRV trajectories to define autonomic endophenotypes; second, multi-omics profiling to identify biomarkers associated with high-risk phenotypes; and third, prospective validation in independent cohorts [49]. This multi-dimensional approach promises to move beyond one-size-fits-all risk stratification toward truly individualized prediction [50].

#### 2.7.3. Novel Therapeutic Strategies Targeting the Autonomic Nervous System

A deeper mechanistic understanding of neural remodeling in AF calls for refined, spatially selective neuromodulatory approaches. Future studies should explore selective cardiac GP mapping and ablation guided by real-time usHRV feedback. The goal is not complete autonomic denervation—which may be proarrhythmic in some contexts—but rather a functional “rebalancing” of sympathetic and vagal tone. We propose that high-density epicardial or endocardial mapping, combined with usHRV-derived metrics before and after each ablation lesion, could identify “dominant” GP sites where focal modulation produces the most favorable autonomic shift. Comparative trials between conventional pulmonary vein isolation alone versus pulmonary vein isolation plus GP-guided modulation, with usHRV as a mechanistic endpoint, are warranted [51].

In parallel, the development of pharmacological or biologic agents targeting specific neurotransmitter receptors with improved cardiac selectivity is a promising avenue. For example, allosteric modulators of M2 muscarinic receptors that fine-tune vagal output without causing bradycardia, or peripherally restricted β2-adrenergic antagonists that mitigate sympathetic overdrive while sparing central nervous system effects, could serve as preventive or adjunctive therapies. Future research should also explore gene therapy approaches—such as viral vector-mediated delivery of neurotrophic factors or chemogenetic tools (e.g., Designer Receptors Exclusively Activated by Designer Drugs)—to remotely control cardiac autonomic neurons. These strategies, though early in development, hold the potential to prevent AF initiation by stabilizing the neural substrate [52].

#### 2.7.4. Methodological Innovation Through Interdisciplinary Collaboration

Current technologies inadequately quantify the dynamic interplay between sympathetic and parasympathetic activities at the level of the intrinsic cardiac nervous system, nor do they resolve their spatiotemporal interactions within the extracardiac neural network. We call for next-generation cardiac neuroimaging techniques—for instance, high-temporal-resolution PET/CT using novel tracers such as [11C]donepezil for parasympathetic nerve imaging or [11C]CGP-12177 for β-adrenergic receptor visualization. Combined with electrocardiogram-gated acquisition, these approaches could map regional autonomic innervation density and its dynamic changes during AF episodes [53].

Simultaneously, real-time usHRV analysis platforms that integrate biosignal processing with closed-loop neuromodulation are needed. Future engineering efforts should focus on developing low-latency, artifact-robust algorithms that can be embedded into implantable loop recorders or wearable defibrillators, enabling automated detection of high-risk autonomic states and triggering preventive interventions (e.g., vagal nerve stimulation or short-acting antiarrhythmic drugs). Achieving these goals requires close collaboration among biomedical engineers, computational scientists, electrophysiologists, and molecular biologists. Establishing open-access, large-scale datasets of long-term usHRV with simultaneously recorded autonomic neuroimaging or neural activity would serve as a catalyst for the field. Only through such cross-disciplinary innovation can we overcome current technical limitations and unlock the full potential of usHRV in both mechanistic research and clinical practice [54].

## 3. Conclusions and Future Perspectives

This review summarises the mechanistic basis and clinical application of ultra-short heart rate variability in AF prediction. Autonomic neural remodelling plays a critical role in the initiation and progression of AF. As a novel analytical tool, usHRV shows promising potential for early AF prediction, providing a new indicator for identifying high-risk populations and facilitating personalised prevention and treatment strategies.

With continued technological development and further research, usHRV is expected to play an increasingly important role in AF prevention, diagnosis, and management. Interdisciplinary collaboration integrating clinical medicine, biomedical engineering, and artificial intelligence may enable the development of more accurate, accessible, and personalised AF prediction and management systems, ultimately improving outcomes for patients with AF.

## Data Availability

No new data were created or analyzed in this study. Data sharing is not applicable to this article.

## Supplementary Materials

The following supporting information can be downloaded at: https://www.mdpi.com/article/10.3390/jcdd13060286/s1, Table S1: Summary of studies examining ultra-short-term heart rate variability for association of AF susceptibility [33,41,45,48,55,56,57,58,59].

## References

[1] Linz D., Ukena C., Mahfoud F., Neuberger H.R., Böhm M. (2014). Atrial autonomic innervation: A target for interventional antiarrhythmic therapy?. J. Am. Coll. Cardiol..

[2] Hanna P., Buch E., Stavrakis S., Meyer C., Tompkins J.D., Ardell J.L., Shivkumar K. (2021). Neuroscientific therapies for atrial fibrillation. Cardiovasc. Res..

[3] Chakraborty P., Farhat K., Po S.S., Armoundas A.A., Stavrakis S. (2023). Autonomic Nervous System and Cardiac Metabolism: Links Between Autonomic and Metabolic Remodeling in Atrial Fibrillation. JACC Clin. Electrophysiol..

[4] Kusayama T., Wan J., Yuan Y., Chen P.S. (2021). Neural Mechanisms and Therapeutic Opportunities for Atrial Fibrillation. Methodist Debakey. Cardiovasc. J..

[5] Shen M.J., Zipes D.P. (2014). Role of the autonomic nervous system in modulating cardiac arrhythmias. Circ. Res..

[6] Manolis A.A., Manolis T.A., Apostolopoulos E.J., Apostolaki N.E., Melita H., Manolis A.S. (2021). The role of the autonomic nervous system in cardiac arrhythmias: The neuro-cardiac axis, more foe than friend?. Trends Cardiovasc. Med..

[7] Hou Y., Zhou Q., Po S.S. (2016). Neuromodulation for cardiac arrhythmia. Heart Rhythm.

[8] Kharbanda R.K., van der Does W.F.B., van Staveren L.N., Taverne Y.J.H.J., Bogers A.J.J.C., de Groot N.M.S. (2022). Vagus Nerve Stimulation and Atrial Fibrillation: Revealing the Paradox. Neuromodulation.

[9] Chen P.S., Chen L.S., Fishbein M.C., Lin S.F., Nattel S. (2014). Role of the autonomic nervous system in atrial fibrillation: Pathophysiology and therapy. Circ. Res..

[10] Carnagarin R., Kiuchi M.G., Ho J.K., Matthews V.B., Schlaich M.P. (2018). Sympathetic Nervous System Activation and Its Modulation: Role in Atrial Fibrillation. Front. Neurosci..

[11] Brundel B., Ai X., Hills M.T., Kuipers M.F., Lip G.Y.H., de Groot N.M.S. (2022). Atrial fibrillation. Nat. Rev. Dis. Primers.

[12] Karatela M.F., Fudim M., Mathew J.P., Piccini J.P. (2023). Neuromodulation therapy for atrial fibrillation. Heart Rhythm.

[13] Wong B., Kuwabara Y., Salavatian S. (2025). Neuromodulation of the Cardiac Autonomic Nervous System for Arrhythmia Treatment. Biomedicines.

[14] Waldron N.H., Fudim M., Mathew J.P., Piccini J.P. (2019). Neuromodulation for the Treatment of Heart Rhythm Disorders. JACC Basic Transl. Sci..

[15] Tan A.Y., Chen P.-S., Chen L.S., Fishbein M.C. (2007). Autonomic nerves in pulmonary veins. Heart Rhythm.

[16] Patterson E., Po S.S., Scherlag B.J., Lazzara R. (2005). Triggered firing in pulmonary veins initiated by in vitro autonomic nerve stimulation. Heart Rhythm.

[17] Huang J., Wu B., Qin P., Cheng Y., Zhang Z., Chen Y. (2023). Research on atrial fibrillation mechanisms and prediction of therapeutic prospects: Focus on the autonomic nervous system upstream pathways. Front. Cardiovasc. Med..

[18] Zhang L., Li B., Wu L. (2025). Heart rate variability in patients with atrial fibrillation of sinus rhythm or atrial fibrillation: Chaos or merit?. Ann. Med..

[19] Shen M.J. (2021). The cardiac autonomic nervous system: An introduction. Herzschrittmacherther. Elektrophysiol..

[20] Castillo-Aguilar M., Mabe-Castro D., Medina D., Núñez-Espinosa C. (2025). Enhancing cardiovascular monitoring: A non-linear model for characterizing RR interval fluctuations in exercise and recovery. Sci. Rep..

[21] Khan A.A., Lip G.Y.H., Shantsila A. (2019). Heart rate variability in atrial fibrillation: The balance between sympathetic and parasympathetic nervous system. Eur. J. Clin. Investig..

[22] Shaffer F., Ginsberg J.P. (2017). An Overview of Heart Rate Variability Metrics and Norms. Front. Public Health.

[23] Yan S.P., Song X., Wei L., Gong Y.S., Hu H.Y., Li Y.Q. (2023). Performance of heart rate adjusted heart rate variability for risk stratification of sudden cardiac death. BMC Cardiovasc. Disord..

[24] Berger M., Pichot V., Solelhac G., Marques-Vidal P., Haba-Rubio J., Vollenweider P., Waeber G., Preisig M., Barthélémy J.C., Roche F. (2022). Association between nocturnal heart rate variability and incident cardiovascular disease events: The HypnoLaus population-based study. Heart Rhythm.

[25] Braffett B.H., El Ghormli L., Martin C., White N.H., Hirsch I.B., Bantle A., Carlson A., Leschek E., Gubitosi-Klug R., Soliman E.Z. (2025). Cardiovascular autonomic neuropathy defined by indices of heart rate variability is associated with cardiovascular disease: A longitudinal cohort study of participants with type 1 diabetes. Cardiovasc. Diabetol..

[26] Tang B., He K., Liu S., Wu Z., Yang C. (2025). Preoperative ECG-assisted feature engineering enhances prediction of new-onset atrial fibrillation after cardiac surgery. Comput. Methods Programs Biomed..

[27] Rai R., Singh V., Ahmad Z., Jain A., Jat D., Mishra S.K. (2024). Autonomic neuronal modulations in cardiac arrhythmias: Current concepts and emerging therapies. Physiol. Behav..

[28] Coumel P. (1996). Autonomic influences in atrial tachyarrhythmias. J. Cardiovasc. Electrophysiol..

[29] Lo L.W., Chiou C.W., Lin Y.J., Chang S.L., Hu Y.F., Tsao H.M., Chao T.F., Li C.H., Chang H.Y., Chung F.P. (2013). Differences in the atrial electrophysiological properties between vagal and sympathetic types of atrial fibrillation. J. Cardiovasc. Electrophysiol..

[30] Thackeray J.T., Bengel F.M. (2016). PET imaging of the autonomic nervous system. Q. J. Nucl. Med. Mol. Imaging.

[31] Shen M.J., Choi E.K., Tan A.Y., Lin S.F., Fishbein M.C., Chen L.S., Chen P.S. (2011). Neural mechanisms of atrial arrhythmias. Nat. Rev. Cardiol..

[32] Hämmerle P., Aeschbacher S., Schlageter V., Coslovsky M., Hennings E., Krisai P., Coduri F., Blum M.R., Rodondi N., Reichlin T. (2024). Heart rate variability and stroke or systemic embolism in patients with atrial fibrillation. Heart Rhythm.

[33] Orini M., van Duijvenboden S., Young W.J., Ramírez J., Jones A.R., Hughes A.D., Tinker A., Munroe P.B., Lambiase P.D. (2023). Long-term association of ultra-short heart rate variability with cardiovascular events. Sci. Rep..

[34] Munoz M.L., van Roon A., Riese H., Thio C., Oostenbroek E., Westrik I., de Geus E.J., Gansevoort R., Lefrandt J., Nolte I.M. (2015). Validity of (Ultra-)Short Recordings for Heart Rate Variability Measurements. PLoS ONE.

[35] Castaldo R., Montesinos L., Melillo P., James C., Pecchia L. (2019). Ultra-short term HRV features as surrogates of short term HRV: A case study on mental stress detection in real life. BMC Med. Inform. Decis. Mak..

[36] Jin K., Guo Z., Qiao Z., Liu M., Yang Y., Xu C. (2024). Agreement between Ultra-Short-Term and Standard Heart Rate Variability Analysis in Resting and Post-Exercise Conditions. Life.

[37] Dekker J.M., Crow R.S., Folsom A.R., Hannan P.J., Liao D., Swenne C.A., Schouten E.G. (2000). Low heart rate variability in a 2-minute rhythm strip predicts risk of coronary heart disease and mortality from several causes: The ARIC Study. Atherosclerosis Risk in Communities. Circulation.

[38] Tsuji H., Larson M.G., Venditti F.J., Manders E.S., Evans J.C., Feldman C.L., Levy D. (1996). Impact of reduced heart rate variability on risk for cardiac events. The Framingham Heart Study. Circulation.

[39] Habibi M., Chahal H., Greenland P., Guallar E., Lima J.A.C., Soliman E.Z., Alonso A., Heckbert S.R., Nazarian S. (2019). Resting Heart Rate, Short-Term Heart Rate Variability and Incident Atrial Fibrillation (from the Multi-Ethnic Study of Atherosclerosis (MESA)). Am. J. Cardiol..

[40] Hillebrand S., Gast K.B., de Mutsert R., Swenne C.A., Jukema J.W., Middeldorp S., Rosendaal F.R., Dekkers O.M. (2013). Heart rate variability and first cardiovascular event in populations without known cardiovascular disease: Meta-analysis and dose-response meta-regression. Europace.

[41] Burma J.S., Graver S., Miutz L.N., Macaulay A., Copeland P.V., Smirl J.D. (2021). The validity and reliability of ultra-short-term heart rate variability parameters and the influence of physiological covariates. J. Appl. Physiol..

[42] Nussinovitch U., Elishkevitz K.P., Kaminer K., Nussinovitch M., Segev S., Volovitz B., Nussinovitch N. (2011). The efficiency of 10-second resting heart rate for the evaluation of short-term heart rate variability indices. Pacing Clin. Electrophysiol..

[43] Barthelemy J.C., Pichot V., Hupin D., Berger M., Celle S., Mouhli L., Bäck M., Lacour J.R., Roche F. (2022). Targeting autonomic nervous system as a biomarker of well-ageing in the prevention of stroke. Front. Aging Neurosci..

[44] Kim S.H., Lim K.R., Seo J.H., Ryu D.R., Lee B.K., Cho B.R., Chun K.J. (2022). Higher heart rate variability as a predictor of atrial fibrillation in patients with hypertension. Sci. Rep..

[45] Tang X., Wu Y., Zhang X., Zhang K., Xie Y., Chao Y., He R., Zhang P. (2025). Association between ultra-short-term heart rate variability of time fluctuation and atrial fibrillation: Evidence from MIMIC-IV. Heart Rhythm O^2^.

[46] Grégoire J.M., Gilon C., Marelli F., Godart P., Bersini H., Carlier S. (2025). Autonomic Nervous System Activity before Atrial Fibrillation Onset as Assessed by Heart Rate Variability. Rev. Cardiovasc. Med..

[47] Vermunicht P., Buyck C., Naessens S., Hens W., Van Craenenbroeck E., Piedrahita Giraldo J.S., Makayed K., Herman S., Laukens K., Roeykens J. (2026). A novel machine learning procedure to detect and remove artefacts in heart rate data obtained from photoplethysmography wearables: A prospective cohort study. Digit Health.

[48] Grégoire J.M., Gilon C., Marelli F., Bersini H., Groben L., Nguyen T., Deruyter B., Godart P., Carlier S. (2025). Short-term atrial fibrillation onset prediction using machine learning. Eur. Heart J. Digit. Health.

[49] Liu L., Yi Y., Yan R., Hu R., Sun W., Zhou W., Zhou H., Si X., Ye Y., Li W. (2024). Impact of age-related gut microbiota dysbiosis and reduced short-chain fatty acids on the autonomic nervous system and atrial fibrillation in rats. Front. Cardiovasc. Med..

[50] Huang F., Zhong Y., Zhang R., Bai W., Li Y., Gong S., Chen S., Zhu T., Chen Y., Rao L. (2024). Cluster Analysis and Ablation Success Rate in Atrial Fibrillation Patients Undergoing Catheter Ablation. Sichuan Da Xue Xue Bao Yi Xue Ban.

[51] Kviesulaitis V., Puodziukynas A., Pauza D.H., Zabiela V., Kazakevicius T., Vaitkevicius R., Diržinauskas E., Semaška V., Strazdas A., Unikaite R. (2017). Heart rate variability after radiofrequency ablation of epicardial ganglionated plexuses on the ovine left atrium. BMC Cardiovasc. Disord..

[52] Shanks J., Herring N. (2013). Peripheral cardiac sympathetic hyperactivity in cardiovascular disease: Role of neuropeptides. Am. J. Physiol. Regul. Integr. Comp. Physiol..

[53] Boutagy N.E., Sinusas A.J. (2017). Recent Advances and Clinical Applications of PET Cardiac Autonomic Nervous System Imaging. Curr. Cardiol. Rep..

[54] Cannard C., Delorme A., Wahbeh H. (2024). HRV and EEG correlates of well-being using ultra-short, portable, and low-cost measurements. Prog. Brain Res..

[55] Tang X., He R. (2025). Association Between Ultra-Short-Term Heart Rate Variability and Atrial Fibrillation in Heart Failure Population: A Retrospective Cohort Study. Heart Lung Circ..

[56] Hillmann H.A.K., Hermans A.N.L., Gawalko M., Mueller-Leisse J., Betz K., Sohaib A., Fung C.H., Pisters R., Lodziński P., Chaldoupi S.-M. (2025). Ultra-short-term heart rate variability using a photoplethysmography-based smartphone application: A TeleCheck-AF subanalysis. Eur. Heart J. Digit. Health.

[57] Salahuddin L., Cho J., Jeong M.G., Kim D. (2007). Ultra short term analysis of heart rate variability for monitoring mental stress in mobile settings. Annu. Int. Conf. IEEE Eng. Med. Biol. Soc..

[58] Nussinovitch U., Elishkevitz K.P., Katz K., Nussinovitch M., Segev S., Volovitz B., Nussinovitch N. (2011). Reliability of Ultra-Short ECG Indices for Heart Rate Variability. Ann. Noninvasive Electrocardiol..

[59] Meehan Z., Shearman S., Shaffer F. (2016). The Promise of Ultra-Short-Term (UST) Heart Rate Variability Measurements. Biofeedback.

