# Supplementary material for: Autonomic Nervous Dysfunction and Ultra-Short-Term Heart Rate Variability in Atrial Fibrillation: Recent Advances in Early Detection
Source: J Cardiovasc Dev Dis. 2026 Jun 22;13(6):286. doi: 10.3390/jcdd13060286 (PMC13301170; doi:10.3390/jcdd13060286)
Supplement: Supplementary file 1 [file jcdd-13-00286-s001.zip › jcdd-4253473-supplementary.pdf]

Supplementary Table S1. Summary of studies examining ultra-short-term heart rate variability for association of AF susceptibility

First author, year	Study aim(s)	Cohort (n, population)	Methodology (recording duration, device, HRV metrics)	Key findings on usHRV and AF / HRV measurement
**Orini M, 2023** (Sci Rep)	To validate usHRV measured using ≤15-s ECGs and investigate its association with AF, MACE, stroke, and mortality	**Validation:** NSHD (n=1,337); **Outcome:** UK Biobank (n=51,628, median age 58)	**Duration:** 15-s, 10-s, 5-s ECGs<br>**Metrics:** RMSSD, SDSD, PHF<br>**Analysis:** Correlation with 6-min HRV, Cox regression<br>**Follow-up:** median 11.5 years	Agreement between 15-s and 6-min HRV was very good for RMSSD and SDSD. Reduced usHRV (<20th percentile) predicted AF (HR 1.17, 95% CI 1.04–1.32), MACE, stroke, and mortality.
**Tang X, 2025** (Heart Rhythm O2)	To investigate the association between usHRV metrics and AF using the MIMIC-IV database	48,416 ICU patients (MIMIC-IV database)	**Duration:** 10-s ECGs<br>**Device:** 12-lead ECG<br>**Metrics:** Time-domain (SDNN, SDSD, RMSSD) and frequency-domain (LF, HF, LF/HF, LFnu, HFnu, TP)<br>**Analysis:** Cox regression, subgroup analysis	Frequency-domain metrics showed greater stability than time-domain metrics. Decreased frequency-domain metrics were significantly associated with increased AF risk [e.g., log(LF/HF): HR 0.79, 95% CI 0.74–0.84]. Associations were stronger in individuals aged ≥55 years.

**Tang X, 2025** (Heart Lung Circ)	To explore the association between usHRV and AF risk in the HFrEF population (retrospective cohort, MIMIC-IV)	2,122 HFrEF patients (MIMIC-IV database)	**Duration:** 10-s ECGs<br>**Device:** 12-lead ECG<br>**Metrics:** SDNN, SDSD, RMSSD, LF, HF, LF/HF, LFnu, HFnu, TP<br>**Analysis:** Multivariate Cox proportional hazards models, Kaplan-Meier curves, E-value analysis<br>**Follow-up:** mean 1.67 years	475 patients (22.4%) developed AF. Decreased usHRV frequency-domain metrics were associated with increased AF risk in fully adjusted models: log(LF): HR 0.88 (0.80–0.96), log(LF/HF): HR 0.82 (0.70–0.95), log(LFnu): HR 0.79 (0.65–0.97), log(HFnu): HR 2.48 (1.42–4.35). Kaplan-Meier curves showed lower usHRV frequency-domain indices correlated with higher AF incidence.
**Hillmann HAK, 2025** (Eur Heart J Digit Health)	To evaluate the feasibility of using PPG-based smartphone recordings for usHRV assessment within a structured mobile health AF management approach	TeleCheck-AF project patients	**Duration:** 1-min PPG recordings<br>**Device:** Smartphone PPG<br>**Metrics:** RMSSD<br>**Analysis:** Inter-recording consistency, correlation with clinical factors	usHRV can be reliably determined in 1-min PPG recordings with high user compliance and high inter-recording consistency. Older age and lower BMI were associated with higher RMSSD. PPG-derived usHRV is feasible for AF management.

**Parsi A, 2021** (Comput Biol Med)	To develop and validate a machine learning model to predict paroxysmal AF onset using HRV features, including short-duration HRV analysis	872 patients with documented paroxysmal AF episodes	**Duration:** short-term HRV analysis preceding AF onset<br>**Device:** Holter ECG<br>**Metrics:** HRV features (time-domain, frequency-domain, nonlinear) combined with fragmentation indices<br>**Analysis:** XGBoost machine learning	The model achieved high prediction accuracy. Key predictive features included short-term vagal activity, HRV fragmentation indices, and nonlinear parameters, highlighting the role of ANS in AF initiation.
**Salahuddin L, 2007** (EMBC)	To determine the minimum recording duration required for valid usHRV estimation of 5-min values under resting and mental stress conditions	24 healthy students (age 22–31)	**Duration:** 10 s to 5 min<br>**Device:** ECG<br>**Metrics:** HR, RMSSD, pNN50, SDNN, LF ms², LF nu, HF ms², HF nu, VLF ms², LF/HF, HTI, TINN<br>**Analysis:** Correlation with 5-min values	HR, RMSSD: 10 s<br>pNN50, HF ms², HF nu, LF/HF, LF nu: 20 s<br>LF ms²: 30 s<br>vLF ms²: 50 s<br>SDNN: 60 s<br>HTI, TINN: 90 s
**Nussinovitch U, 2011** (Ann Noninvas Electrocardio)	To evaluate the reliability of usHRV indices as surrogates for 5-min short-term HRV	70 healthy volunteers (age 42.5±16.1)	**Duration:** 10 s, 1 min<br>**Device:** ECG<br>**Metrics:** RMSSD, SDNN<br>**Analysis:** Correlation with 5-min recordings	Ultra-short-term RMSSD (10 s, 1 min) values correlated acceptably with 5-min RMSSD; SDNN did not achieve acceptable correlation with 5-min SDNN.

**Shaffer F, 2016** (Biofeedback)	To determine the minimum recording duration for usHRV metrics to validly estimate 5-min HRV values in healthy young adults	38 healthy university students (age 18–23)	**Duration:** 10, 20, 30, 60, 90, 120, 180, 240 s<br>**Device:** ECG<br>**Metrics:** HR, SDNN, RMSSD, pNN50, TINN, HTI, LF ms², LF nu, HF ms², HF nu, LF/HF, VLF ms², SD1, SD2, DFA α1, DFA α2, SampEn, ShanEn, DET<br>**Analysis:** Correlation with 5-min values (criterion: r≥0.90)	HR: 10 s; pNN50: 60 s; TINN, LF ms², SD1, SD2: 90 s; HTI, DFA α1: 120 s; LF nu, HF ms², HF nu, LF/HF, SampEn, DFA α2, DET: 180 s; ShanEn: 240 s; VLF ms²: 270 s; CD could not be estimated from any UST epoch.
**Burma JS, 2021** (J Appl Physiol)	To systematically evaluate the concurrent validity, within-day reliability, and between-day reliability of usHRV compared to short-term HRV, and to examine the influence of physiological covariates	36 healthy adults (18 males, age 26±5 years)	**Duration:** 30, 60, 120, 180, 240 s extracted from 300-s standing recordings<br>**Device:** 3-lead ECG<br>**Metrics:** HR, SDNN, RMSSD, pNN50, absolute/relative LF, absolute/relative HF, LF/HF<br>**Analysis:** Bland-Altman, repeated measures ANOVA, linear regression, ICC, CoV	HR showed acceptable validity/reliability with ≥60 s; time-domain metrics (SDNN, RMSSD) required ≥240 s; relative frequency-domain metrics required 300 s. Shorter usHRV (≤180 s) showed lower validity/reliability and greater susceptibility to physiological confounders (respiratory rate, $PETCO_2$, MAP). Absolute frequency-domain metrics and pNN50 were highly variable and not recommended in the upright position.
